# An Entropy-based gene selection method for cancer classification using microarray data

**DOI:** 10.1186/1471-2105-6-76

**Published:** 2005-03-24

**Authors:** Xiaoxing Liu, Arun Krishnan, Adrian Mondry

**Affiliations:** 1 Bioinformatics Institute, 30, Biopolis Street, #07-01, (S) 138671, Singapore

## Abstract

**Background:**

Accurate diagnosis of cancer subtypes remains a challenging problem. Building classifiers based on gene expression data is a promising approach; yet the selection of non-redundant but relevant genes is difficult.

The selected gene set should be small enough to allow diagnosis even in regular clinical laboratories and ideally identify genes involved in cancer-specific regulatory pathways. Here an entropy-based method is proposed that selects genes related to the different cancer classes while at the same time reducing the redundancy among the genes.

**Results:**

The present study identifies a subset of features by maximizing the relevance and minimizing the redundancy of the selected genes. A merit called *normalized mutual information *is employed to measure the relevance and the redundancy of the genes. In order to find a more representative subset of features, an iterative procedure is adopted that incorporates an initial clustering followed by data partitioning and the application of the algorithm to each of the partitions. A leave-one-out approach then selects the most commonly selected genes across all the different runs and the gene selection algorithm is applied again to pare down the list of selected genes until a *minimal *subset is obtained that gives a satisfactory accuracy of classification.

The algorithm was applied to three different data sets and the results obtained were compared to work done by others using the same data sets

**Conclusion:**

This study presents an entropy-based iterative algorithm for selecting genes from microarray data that are able to classify various cancer sub-types with high accuracy. In addition, the feature set obtained is very compact, that is, the redundancy between genes is reduced to a large extent. This implies that classifiers can be built with a smaller subset of genes.

## Background

DNA microarrays have become ubiquitous in analyzing the expression profiles of genes in the hope to distinguish between various disease types, such as discriminating between various cancer sub-types. Differential expression of genes is analyzed statistically and genes are assigned to various classes which may (or not) enhance the understanding of underlying biological processes. Alternatively, a reduced set of genes may be singled out and used as biomarkers for diagnosis and prognosis.

Microarray data is typically used both to discover new classes as well as in class prediction. Discovery of new classes [1-4] is usually achieved with the help of clustering techniques such as hierarchical clustering [5], k-means clustering [6] and self organizing maps (SOM) [7]. Class prediction, involving the assignment of labels to samples based on their expression patterns, is typically based on statistical or supervised machine learning methods. These range from the application of simple techniques such as nearest neighbor algorithms [8] to classical methods such as linear discriminant analysis [9] to more advanced techniques such as neural networks [10], support vector machines [11-13], fuzzy logic [14] and decision trees [15]. The challenge in dealing with microarray data lies in the fact that there are orders of magnitude differences between the number of samples (typically less than a hundred) and the number of genes (typically tens of thousands) that are studied. The measurements also typically contain both measurement noise as well as systemic noise. This could have a significant impact on classification accuracy. Classification must therefore be preceded by a step known as feature selection where a subset of relevant features is identified.

There are a number of advantages to feature set selection. The first lies in reducing the cost of clinical diagnosis. It is much cheaper to focus only on the expression of a few genes rather than on thousands of genes for diagnosis [16]. Feature set selection can also lead to a reduction in computational cost as a result of a reduction in problem dimensionality. Furthermore, feature set selection often gives rise to a much smaller and a more compact gene set. This could make it easier to identify genes of particular importance to the problem under study. Moreover, given the disparity in the magnitudes of the numbers of genes and samples, it is difficult to justify the development of a classifier based on a gene set where the number of genes is greater than the number of samples.

One way to categorize feature set selection approaches is to classify them as either filter (such as those based on statistical tests such as *t*-test, *F*-test etc.) or wrapper [17] methods. These methods have the advantage of having very low computational complexity as well as better generalization potential since they are uncorrelated to the learning method.

Wrapper type approaches are those in which the feature selection method is bundled together with the learning method. This implies that the usefulness of a feature is validated by the estimated accuracy of the learning method. In consequence, often, a small subset of the feature set with very high prediction accuracy can be obtained because the characteristics of the features match well with the characteristics of the learning method.

Another way of categorizing feature set selection approaches is as univariate or multivariate [18]. Univariate methods [1,19] consider the contributions of individual genes to the classification independently. In contrast multivariate methods such as recursive feature elimination (RFE) [12], leave one out (LOO) method [13], mutual information based approaches [20] etc., measure the relative contribution of a gene to the classification by taking the effect of other genes into consideration at the same time.

A serious deficiency of currently used multivariate approaches for feature set selection is that they are based on selecting genes which are maximally relevant with respect to the classes. The problem with this approach is that there might still be genes among the selected set that are heavily correlated with each other and thus leading to a redundancy in the selected feature set. Ding et. al. [20] have used mutual information for gene selection that has maximum relevance with minimal redundancy by solving a simple two-objective optimization.

In the study presented here, a similar approach has been followed for feature set selection by trying to maximize the relevance and minimize the redundancy of the selected genes. However, *normalized mutual information *has been used instead of mutual information. In addition, both Battiti's greedy selection algorithm [21] as well as a simulated annealing based approach [22] have been used. In order to find a more representative subset of features, an iterative procedure was adopted that incorporates an initial clustering followed by data partitioning and the application of the algorithm to each of the partitions. A leave-one-out approach then selects the most commonly selected genes across all the different runs and the gene selection algorithm is applied again to pare down the list of selected genes until a *minimal *subset that gives a satisfactory accuracy of classification is obtained. The algorithm was applied to three different data sets and the results obtained were compared to work done by others using the same data sets. Additionally the algorithm was also compared to work done by Ding and Peng [20] for three different datasets.

## Results

### Datasets

Three public microarray data sets were used to assess the performance of the algorithm.

#### SRBCT data

This data set includes 88 cDNA arrays for 63 training samples and 25 test samples from [10]. All samples were combined together and the 5 non-SRBCT samples were removed. The data set consists of four types of tumors in childhood, including Ewing's sarcoma (EWS), rhabdomyosarcoma (RMS), neuroblastoma (NB), and Burkitt lymphoma (BL). After filtering by [10], 2308 genes remained in the data set. The data was transformed to natural logarithmic values. Finally, each sample was also standardized to zero mean and unit variance.

#### Breast cancer data

This data set contains expression levels of 7129 genes in 49 breast tumor samples from [23]. The samples were classified according to their estrogen receptor (ER) status. 25 samples were ER positive while the other 24 samples were ER negative. In the pre-processing procedure, the data was thresholded with a floor of 100 and a ceiling of 16000 Affymetrix intensity units. Then those genes with  ≤ 5 or *max - min *≤ 500 were excluded. The filtered data was transformed to base 10 logarithmic values. Finally, each sample was standardized to zero mean and unit variance.

#### Colon cancer data

This data set contains expression levels of 40 tumor and 22 normal colon tissues. Only the 2000 genes with the highest minimal intensity were selected by [24]. The data waspre-processed by transforming the raw intensities to base 10 logarithmic values and standardizing each sample to zero mean and unit variance.

### Results

**Figure 1:**
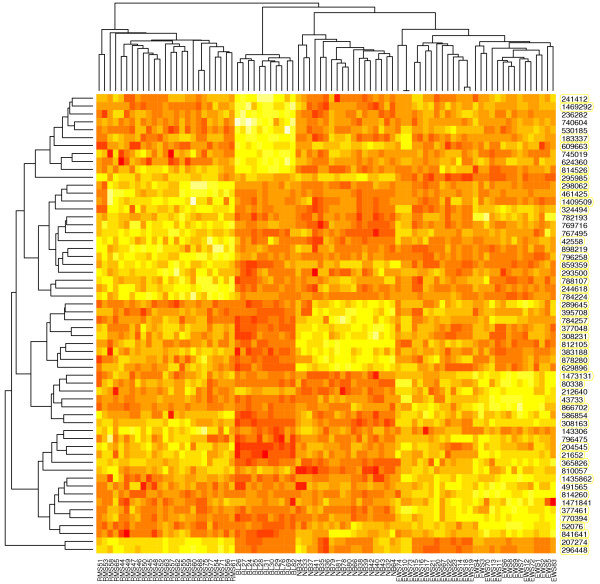
Clustering dendrogram of SRBCT data – First Iteration.

The results of the application of the full algorithm using both the greedy selection algorithm as well as the simulated annealing algorithm for solving Problem 2 are shown in Table 1. The associated clustering dendrograms are shown in Figures 1, 3 and 5, respectively. For all the dendrograms, the samples are presented along the x-axis with the gene-set along the y-axis. *Orange *reflects up-expression while *yellow *represents no or little expression.

The results for SRBCT were the best with a 100% accuracy obtained. The number of genes selected in this case was 58 as opposed to the 96 genes selected by Khan *et al*. [10]. It is interesting to note that when the binary optimization algorithm was used to select genes for the SRBCT data, 50 of the genes selected were the same as those selected with the greedy algorithm. The accuracy rate for breast cancer data was similar for both cases with about 5 samples being misclassified. The final gene set for this data set contained 31 genes. For colon cancer data, there were 6 mis-classifications, with an overall accuracy rate of 90.3%. There were 29 genes in the final selected gene set.

**Figure 2:**
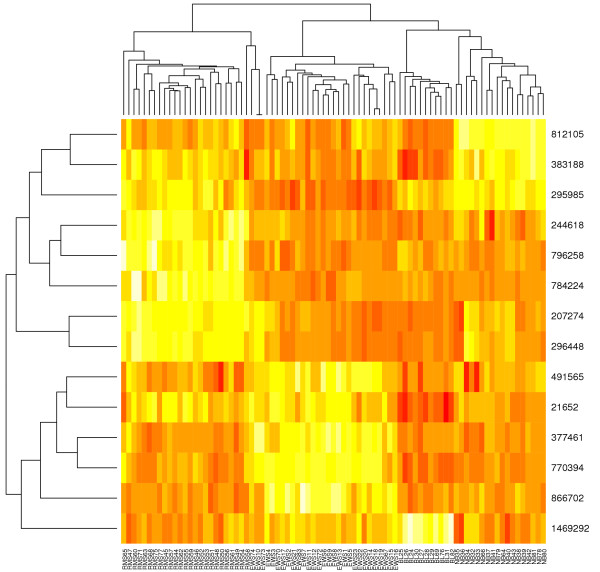
Clustering dendrogram of SRBCT data – Reduced Feature Set.

**Figure 3:**
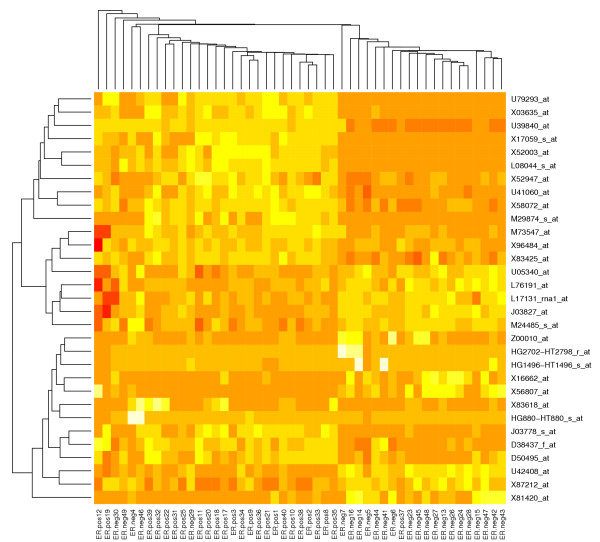
Clustering dendrogram of breast cancer data – First Iteration.

**Figure 4:**
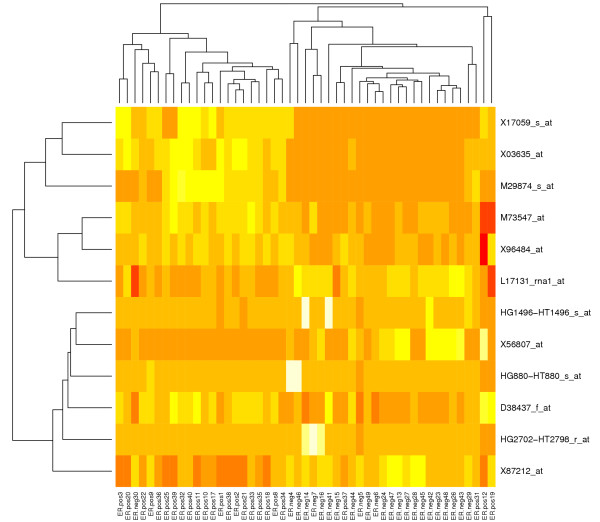
Clustering dendrogram of breast cancer data – Reduced Feature Set.

**Figure 5:**
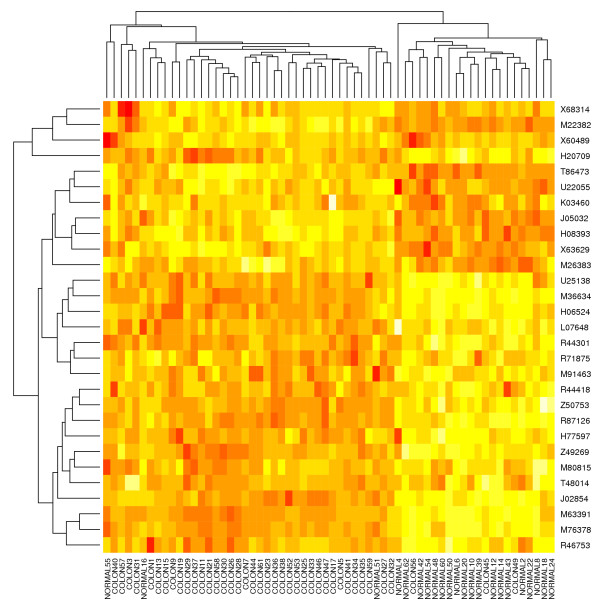
Clustering dendrogram of colon cancer data – First Iteration.

**Figure 6:**
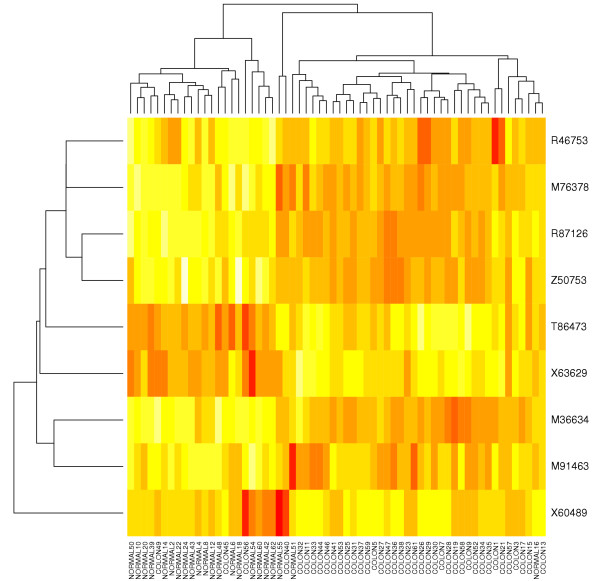
Clustering dendrogram of colon cancer data – Reduced Feature Set.

There seems to be no quantitative or qualitative difference when using the greedy selection or the binary optimization algorithm. Moreover, since the simulated annealing procedure requires an inordinate amount of computation time (of the order of days) as compared to the greedy selection algorithm (of the order of a couple of hours), the iterative procedure was implemented with the greedy algorithm. The iterative approach shown in Figure 8 was used for all three data sets and the clustering dendrograms with the reduced feature sets are shown in Figures 2, 4 and 6 respectively. It is interesting to note that the classification accuracy is not affected by using a much reduced feature set. In fact, for colon cancer data, the accuracy improved to 91.9%.

One of the main concerns while carrying out a multi-objective optimization is the presence of the weight factor *β*. The selection of *β *is usually heuristic. Battiti suggested in [21] that the value of *β *between 0.5 and 1.0 is appropriate for most cases. The effect of changing *β *was studied by changing its value from 0 to 1 in steps of 0.2. using the colon cancer data set and the classification accuracy calculated (Table 2). A value of (0.5 – 1.0) for *β *seems appropriate. Also, the order of selection of the first 10 genes was examined (Table 3). It appears that varying *β *does affect the gene selection order to a certain extent. For example, comparing the gene selection orders for *β *= 0.6, 0.8 reveals that genes 267 and 513 swap places while genes 1256 (for *β *= 0.6) and 1727 (for *β *= 0.8) are not common to both cases. However, it must be kept in mind that the selection order in this case is *not *indicative of the relative importance of the genes since a greedy algorithm is being used.

We also compared our methodology to that of Ding and Peng [20] for three different datasets. The first dataset is the colon cancer dataset [24]. The second dataset is the leukemia dataset [1]. The third and final dataset used was the NCI dataset [25]. The results are tabulated in Table 4. As can be observed, the Uncertainity-based (UB) method (our method) seemed to do better than the DP (Ding and Peng) method for the colon dataset. On the other hand, for the leukemia dataset, DP proved superior to our method. For the NCI dataset, both methods performed poorly with the DP method having a slight edge. It must however be noted that the NCI dataset consists of 9 classes and only 60 samples. As a result, classifying the dataset with a very small sample size into 9 different classes and using only 15 genes is very difficult.

A further difficulty in comparing different methodologies lies in the fact that the initial pre-processing step could also play a role in classification accuracies. In the absence of a uniformity of preprocessing of the datasets, it is difficult to draw general conclusions regarding the relative performances of two different methodologies.

As commonly observed when analytical algorithms are compared, the performance shows mixed results. While Ding and Peng algorithm outperformed the one presented here (see table 4), it should be noted that the description of methods in their article did not allow us to compare both algorithms on equal terms as no gene ranking was provided, and thus the biological significance of their findings could not be assessed.

A comparison between the accuracies obtained by the original papers (from which the datasets were obtained) and our method is given in Table 5. The list of genes selected for each dataset and their ranks in the original papers are given in the supplementary file.

## Discussion

The details of the selected genes and the comparison with the original data are listed in the supplementary material for all three data sets. This section presents a discussion of the comparison of genes selected by the algorithm presented in this work with those presented in earlier work (or as in the case of Breast cancer and SRBCT data, in the original work).

### SRBCT data set

There were a total of 41 genes that overlapped between the selection methods presented in this work and those by [10]. The common genes were from all rank levels of the original method. Left out genes coded often, but not always for proteins from a functional system similar to those still selected here, as in the case of no. 233721 insulin-like growth factor binding protein (not selected here) and no. 296448 (insulin- like growth factor 2) and 207274 (insulin-like growth factor 2, exon 7 and additional ORF), which were selected by both methods. Interestingly, two viral oncogene sequences were not selected (nos. 417226 and 812965, v-myc avianmyelocytomatosis viral oncogene homologs), nor were some extra- cellular matrix associated genes (nos: 122159 and 809901, collagens type III and XV) both without replacement from similar genes. The seventeen newly selected genes that were not part of the original selection come from various functional systems. Of interest here is that while the original gene no. 245330 (Human krueppel-related zinc finger protein H-plk) was left out, gene no. 767495 (GLI-Krueppel family member GLI3) was newly selected. Such "nuclear localization signals" have been shown to be involved in processes determining proper nuclear localization [26], but may also be determinants of progression towards cancer [27].

### Breast cancer data set

Out of the 31 genes selected here, 16 were not selected in the original publication [23], which selected 60 genes. The 45 genes not selected by the present method covered a large variety of physiological functions, without a specific pattern becoming obvious. Two genes linked to the ILGF were left out (no: s37730 and m62403), with no replacement. ILGF is linked to the development of a number of cancers (review in [28]). The fact that ILGF-linked genes are left out here may be discussed in two diametrically opposite ways. For once, leaving these genes out of the classification set may cause an oversight of the tissue's potential to induce further cancerous growth. More likely, though, it seems like whatever physiological role these genes play in the tissue, they do not contribute to distinguishing between various types of cancer.

### Colon cancer data set

Contrary to the other two test sets, in the case of colon cancer, the original publication did not rank the gene set retrieved, so that a direct comparison of results was not possible. The same dataset, however, has been re- analyzed previously by Silvio Bicciato [29], using an auto associative neural network model, which yielded a ranked gene list. With the exception of Tetraspan-1, which heads the rank list with a weight of 0.9391, the top genes found by Bicciato for the reconstruction of the normal class concur with the rank list presented here, while only one gene (Heat shock 60 kD protein 1) is selected by both methods when compared to the gene list in [29] for the reconstruction of the tumor class. This tetraspan family of proteins is involved in cell adhesion processes at the gap junctions and one related protein was enhanced in highly metastatic gastric cancer [30].

## Conclusion

Compared to the classification methods described in the original articles or previous third party analysis, the algorithm described here compares favorably in its capacity to select small sets of genes that distinguish between various cancer types. The observation that it leaves out several genes known to be involved in cancer development may indicate that this method's advantage lies more in good classification, but not in the detection of new dysfunctional regulatory mechanisms.

Although preliminary results using a greedy selection algorithm are encouraging, additional work needs to be done in order to develop alternative methodologies for multi-objective optimization that can select a more optimal and representative set of genes for discriminating between various cancer sub-types.

## Methods

Algorithms for microarray data analysis typically focus on obtaining a set of genes that can distinguish between the different classes in a given sample set. Thus, the primary concern is to ensure the relevance of the genes to the classes under consideration.

Given a microarray data set with *m *samples belonging to *k *known classes and *n *genes, we want to select out those genes which are able to predict the differences in the gene expression patterns in different sample classes. Define ; |*c*| = *k*, as the vector labeling the classes of samples and ; *i *∈ *n *as the gene expression profile of gene *i*. Let  be the feature set of all genes and let *S *be the set of selected genes. Then, the feature set selection problem can be defined as follows:

### Problem 1

*Select a set S of genes, S *⊂ * such that ∀ gene s *∈ *S the relevance of s with **is maximized*.

However, the feature set of genes selected will contain a number of redundant genes with sometimes little relevance to the classes. This is due to the fact that the presence of genes that are closely related to each other imply that there is a possibility of genes orthoganal to those in the selected set being left out of the final feature set. Moreover, the presence of genes with little relevance to the classes leads to a reduction in the "useful information".

Ideally, selected genes should have high relevance with the classes while the redundancy among the selected genes is low. Most previous studies emphasized the selection of highly relevant genes. Ding et. al. [20] addressed the issue of the redundancies among the selected genes. The genes with high relevance are expected to be able to predict the classes of the samples. However, the prediction power is reduced if many redundant genes are selected. In contrast, a feature set that contains genes not only with high relevance with respect to the classes but with low mutual redundancy is more effective in its prediction capability.

### Problem formulation

To assess the effectiveness of the genes, both the relevance and the redundancy need to be measured quantitatively. An entropy based correlation measure is chosen here. According to Shannon's information theory [31], the entropy of a random variable *X *can be defined as:



Entropy measures the uncertainty of a random variable. For the measurement of the interdependency of two random variables *X *and *Y*, some researchers [20,21] used mutual information, which is defined as:

*I*(*X*, *Y*) = *H*(*X*) + *H*(*Y*) - *H*(*X, Y*)   (2)

In order to ensure that different values are comparable and have similar effects, *normalized mutual information *is used as a measure and is defined as:



*U*(*X, Y*) is symmetrical and ranges from 0 to 1, with the value 1 indicating that the knowledge of one variable completely predicts the other (high mutual relevance) while the value 0 indicates that *X *and *Y *are independent (low mutual relevance).

The mutual relevance between  and  can then be modeled by *U *() while the dependency between two genes is *U *().

The total relevance of all selected genes is given by



The total redundancy among the selected genes is given by



Therefore, the problem of selecting genes can be reformulated as follows:

### Problem 2

*Select a set S of genes, S *⊂ *such that ∀ g_i _∈ S, the total relevance of all the selected genes with *, *J*_1_, *is maximized while the total relevance among all the selected genes g_i _∈ S, J*_2_, *is minimized*.

This is a two-objective optimization problem. To solve it, a simple way is to combine these two objectives into one:



where *β *is a weight parameter.

subsection* Algorithm

**Figure 7:**
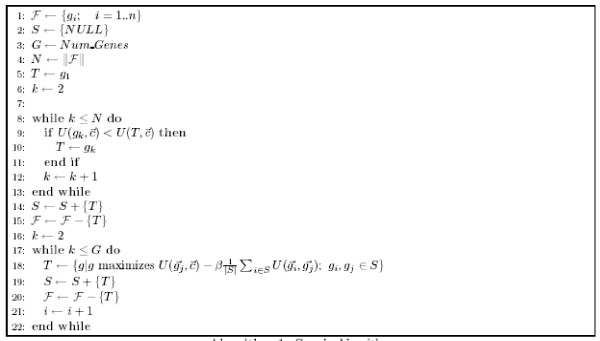
Greedy Algorithm.

To solve the above problem, Battiti [21] proposed a greedy algorithm. The procedure can be described as follows (see Figure 7):

1. Initialization: *F *← *allgenes, S *← ∅.

2. First gene: select gene *i *that has highest relevance *U *(). *g*_*i *_∈ *S*, *F *\ *i*.

3. Remaining genes: From *F*, select gene *j *that maximizes .

4. Repeat the above step until the desired number of genes are obtained.

The maximization problem (6) can also be re-formulated into a binary optimization problem. Let *x*_*i *_be a binary variable with value 1 for selecting gene *i *while value 0 for not. Thus, Equation (6) can be rewritten into:



It can be further rewritten into matrix form:

max *U*_*c*_^*T*^*x *- *βx*^*T*^*U*_*p*_*x*   (8)

where *U*_*c *_is the relevance vector, *U*_*p *_is matrix of pairwise redundancy.

Beasley et al. [32] discussed several heuristic algorithms to solve such binary quadratic programming problems. A heuristic simulated annealing method was employed to solve the problem. The pseudo codes of simulated annealing can be obtained from [32].

There are however limitations to both approaches. There is a possibility that the solution obtained for Problem 2 can lead to a local optimum. This could result in a sub-optimal feature set thereby affecting the prediction accuracy. In order to expand the search space, an iterative procedure was adopted. The data was initially clustered and partitioned into *K *groups, *C*_1_, *C*_2_,..., *C*_*K *_by using k-means clustering. The idea was to group genes with similar expression patterns together. The greedy or heuristic simulated annealing procedure was then applied to select a subset of genes, *S*_*k*_, from each partition, *k*, such that the selected genes had low mutual relevance with respect to each other while at the same having maximal relevance with the different classes. The genes selected from each subset are then combined to obtain a single gene set, that is, *S *= *S*_1 _⋃ *S*_2 _⋃ *S*_3_,..., ⋃*S*_*K*_.

**Figure 8:**
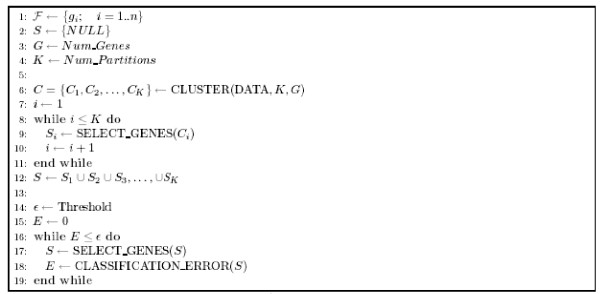
**Optimal Feature Set Selection Algorithm**. The function CLUSTER uses the k-means clustering approach to partition the initial gene set into the desired number of partitions *K*, with *G *genes in each partition. *K *and *G *are user-specified. The function SELECT_GENES uses either the greedy approach (Figure 7) or the heuristic simulated annealing approach to solve Problem 2. The function CLASSIFICATION_ERROR uses kNN classification method to assess the discriminant power of the selected genes and returns the classification error.

The final set of genes is selected by carrying out a leave-one-out cross validation (LOOCV). For each run, one sample is held out for testing whilei the remaining *N *- 1 samples are used to train the classifier. The genes are selected by the algorithm using the training samples and then are used to classify the testing sample. The overall accuracy rate is calculated based on the correctness of the classifications of each testing sample. In order to get a deeper understanding of the selected genes, those genes found in common for all the *N *different runs of the LOOCV experiment are finally listed out for further investigation. The process of gene selection is repeated by selecting a subset of genes from this feature set, that gives a classification error that is below a user defined threshold *ε*. Nearest neighborhood (k-NN) classification method is used to assess the discriminant power of the selected genes by the method. The process is stopped when the error becomes greater than *ε*. The full algorithm is presented in Figure 8.

## Authors' contributions

LXX was responsible for the development and implementation of the algorithm as well as for writing parts of the paper. AK was involved in algorithm development as well as in writing the manuscript. AM was responsible for the analysis of the results as well as manuscript preparation. All authors read and approved the manuscript.

## Supplementary Material

Additional File 1 Selected Genes for All Datasets. The file contains the list of selected genes for each of the three datasets used in this study as well as the corresponding ranks of those selected genes in the original papers. Click here for file

## Figures and Tables

**Table 1:** Classification accuracies and the number of selected genes for the two different optimizationmethods (Greedy and Simulated Annealing (SA)). For Greedy selection, the accuracies as well as the number of genes selected in iterations 1 and 2 are shown. The reported accuracies are LOOCV accuracies while the number of genes is the smaller subset common to all LOOCV experiments.

Algorithm	Colon		Breast		SRBCT	
	
	% Acc	# Genes	% Acc	# Genes	% Acc	# Genes
Greedy	90.3/91.9	29/9	89.8/89.8	31/12	100/100	58/14
SA	87.1/-	26/-	89.8/-	44/-	100/-	58/-

**Table 2:** Effect of varying *β *on classification accuracy. The effect of varying *β *was studied for the colon cancer data set. A value of between 0.5 – 1 as suggested by Battiti [21] seems appropriate.

*β*	0.0	0.2	0.4	0.6	0.8	1.0
accurate	87.1%	88.7%	90.3%	90.3%	90.3%	90.3%

**Table 3:** Effect of varying *β *on the selection order of genes. The first ten genes selected for each value of *β *are shown here. The numbers correspond to the gene numbers for the colon cancer data set. Varying *β *does seem to affect the order in which the genes are selected. However, selection order is not indicative of the relative importance of genes since a greedy-selection method is being used.

*β*	g^1^	g^2^	g^3^	g^4^	g^5^	g^6^	g^7^	g^8^	g^9^	g^10^
0.0	377	267	765	493	1582	513	1635	1671	245	780
0.2	377	267	1582	513	765	493	1635	1671	780	1423
0.4	377	1582	267	513	493	765	1635	1671	780	1491
0.6	377	1582	267	513	1491	493	1635	765	1671	1256
0.8	377	1582	513	267	1491	1727	493	1635	1671	765
1.0	377	1582	1491	513	267	1727	1244	1256	1671	1873

**Table 4:** Classification accuracies and the number of selected genes for the two different mutual information based methodologies (Uncertainity based (UB) and Ding and Peng's (DP)). The accuracies as well as the number of genes selected in iterations 1 and 2 respectively are shown for the UB method while the accuracies and genes selected for two different runs are shown for the DP case. For both methodologies, the accuracies reported are LOOCV accuracies.

Algorithm	Colon		Leukemia		NCI	
	
	% Acc	# Genes	% Acc	# Genes	% Acc	# Genes
UB (ours)	90.3/91.9	29/9	80.6/76.4	21/5	57.6/52.5	59/15
DP	75.8/91.9	50/20	98.6/100	50/10	73.3/61.7	50/20

**Table 5:** Classification Results of Original Papers

Dataset	Colon	Breast	SRBCT
Accuracy (original)	only clustering	89.47	100
Accuracy (UB)	91.9	89.8	100
