# Supplementary material for: An Entropy-based gene selection method for cancer classification using microarray data
Source: BMC Bioinformatics. 2005 Mar 24;6:76. doi: 10.1186/1471-2105-6-76 (PMC1087831; doi:10.1186/1471-2105-6-76)
Supplement: Additional File 1 — Selected Genes for All Datasets. The file contains the list of selected genes for each of the three datasets used in this study as well as the corresponding ranks of those selected genes in the original papers. [file 1471-2105-6-76-S1.pdf]

Selected Genes for All Datasets

March 22, 2005

Table 1: Selected Genes Comparison for SRBCT Data Set

Image ID	Gene	Rank in [2]
296448	insulin-like growth factor 2 somatomedin A)	1
207274	Human DNA for insulin-like growth factor II (IGF-2); exon 7 and additional ORF	2
841641	cyclin D1 (PRAD1: parathyroid adenomatosis 1)	3
365826	growth arrest-specific 1	4
770394	Fc fragment of IgG, receptor, transporter, alpha	6
244618	ESTs	7
43733	glycogenin 2	9
295985	ESTs	10
629896	microtubule-associated protein 1B	11
866702	protein tyrosine phosphatase, non-receptor type 13 (APO-1/CD95 (Fas)-associated phosphatase)	15
377461	caveolin 1, caveolae protein, 22kD	18
52076	olfactomedinrelated ER localized protein	19
308163	ESTs	21
812105	transmembrane protein	22
183337	major histocompatibility complex, class II, DM alpha	23
298062	troponin T2, cardiac	25
204545	ESTs	28
383188	recoverin	29
289645	amyloid beta (A4) precursor-like protein 1	32
324494	heat shock 27kD protein 2	33
1473131	transducin-like enhancer of split 2, homolog of Drosophila E(sp1)	35
878280	collapsin response mediator protein 1	38
609663	protein kinase, cAMP-dependent, regulatory, type II, beta	41
461425	MYL4	42
1469292	pim-2 oncogene	43
1409509	troponin T1, skeletal, slow	48
788107	amphiphysin-like	49
308231	Homo sapiens incomplete cDNA for a mutated allele of a myosin class I, myh-1c	53
21652	catenin (cadherin-associated protein), alpha 1 (102kD)	55
241412	E74-like factor 1 (ets domain transcription factor)	58

Continued on next page

Table 1 – continued from previous page

**Image ID**	**Gene**	**Rank in [2]**
814260	follicular lymphoma variant translocation 1	60
859359	quinone oxidoreductase homolog	61
80338	selenium binding protein 1	63
784224	fibroblast growth factor receptor 4	68
1435862	antigen identified by monoclonal antibodies 12E7, F21 and O13	73
377048	Homo sapiens incomplete cDNA for a mutated allele of a myosin class I, myh-1c	74
784257	kinesin family member 3C	76
42558	glycine amidinotransferase (L-arginine:glycine amidinotransferase)	77
814526	ESTs	78
395708	DPYSL4	80
796258	sarcoglycan, alpha (50kD dystrophin-associated glycoprotein)	89
624360	proteasome (prosome, macropain) subunit, beta type, 8 (large multifunctional protease 7)	-
767495	GLI-Kruppel family member GLI3 (Greig cephalopolysyndactyly syndrome)	-
796475	ESTs, Moderately similar to skeletal muscle LIM-protein FHL3 [H.sapiens]	-
491565	Cbp/p300-interacting transactivator, with Glu/Asp-rich carboxy-terminal domain, 2	-
530185	CD83 antigen (activated B lymphocytes, immunoglobulin superfamily)	-
143306	lymphocyte-specific protein 1	-
745019	EH domain containing 1	-
769716	neurofibromin 2 (bilateral acoustic neuroma)	-
782193	thioredoxin	-
236282	Wiskott-Aldrich syndrome (ecezema-thrombocytopenia)	-
810057	cold shock domain protein A	-
586854	ESTs	-
898219	mesoderm specific transcript (mouse) homolog	-
740604	interferon stimulated gene (20kD)	-
212640	Rho GTPase activating protein 4	-
293500	ESTs	-
1471841	ATPase, Na+/K+ transporting, alpha 1 polypeptide	-

Note: - means not being selected in the list

Table 2: Selected Genes Comparison For Breast Cancer Data Set

**Accession NO**	**Gene**	**Rank in [3]**
X52003	H.sapiens pS2 protein gene	1
X03635	Human mRNA for oestrogen receptor	2

Continued on next page

Table 2 – continued from previous page

Accession NO	Gene	Rank in [3]
M29874	Human cytochrome P450-IIB (hIIB1) mRNA	3
L08044	Human intestinal trefoil factor mRNA	4
U79293	Human clone 23948 mRNA sequence	6
J03778	Human microtubule-associated protein tau mRNA	7
X58072	Human hGATA3 mRNA for trans-acting T-cell specific transcription factor	9
X17059	Human NAT1 gene for arylamine N-acetyltransferase	12
U39840	Human hepatocyte nuclear factor-3 alpha (HNF-3 alpha) mRNA	16
X87212	H.sapiens mRNA for cathepsin C	19
U41060	Human breast cancer, estrogen regulated LIV-1 protein (LIV-1) mRNA	25
D38437	Human DNA mismatch repair mRNA	27
M24485	Homo sapiens (clone pHGST-pi) glutathione S-transferase pi (GSTP1) gene	31
U05340	Human p55CDC mRNA	41
X83425	H.sapiens LU gene for Lutheran blood group glycoprotein	45
X81420	H.sapiens mRNA for hHKb1 protein	-
L76191	Homo sapiens interleukin-1 receptor-associated kinase (IRAK) mRNA	-
HG1496-HT1496	Adrenal-Specific Protein Pg2	-
X16662	Human mRNA for vascular anticoagulant-beta (VAC-beta)	-
U42408	Human ladinin (LAD) mRNA	-
M73547	Human polyposis locus (DP1 gene) mRNA	-
X83618	H.sapiens mRNA for 3-hydroxy-3-methylglutaryl coenzyme A synthase	-
Z00010	H.sapiens germ line pseudogene for immunoglobulin kappa light chain leader peptide and variable region (subgroup V kappa I).	-
D50495	Human mRNA for transcription elongation factor S-II, hS-II-T1	-
X96484	H.sapiens mRNA for DGCR6 protein	-
J03827	Y box binding protein-1 (YB-1) mRNA	-
X52947	Human mRNA for cardiac gap junction protein	-
HG2702-HT2798	Serine/Threonine Kinase	-
X56807	Human DSC2 mRNA for desmocollins type 2a and 2b	-
HG880-HT880	Mucin 6, Gastric	-
L17131	Human high mobility group protein (HMG-I(Y)) gene exons 1-8	-

Note: - means not being selected in the list

Table 3: Selected Genes Comparison For Colon Cancer Data Set

Accession NO	Gene	Weight in [1]
M63391	Human desmin gene	0.5182

Continued on next page

Table 3 – continued from previous page

Accession NO	Gene	Weight in [1]
M76378	Human cysteine-rich protein (CRP) gene	0.4880
R46753	CYCLIN-DEPENDENT KINASE INHIBITOR 1 (Homo sapiens)	0.4450
J02854	MYOSIN REGULATORY LIGHT CHAIN 2, SMOOTH MUSCLE ISOFORM (HUMAN)	0.2912
M22382	MITOCHONDRIAL MATRIX PROTEIN P1 PRECURSOR (HUMAN)	0.1850
Z50753	H.sapiens mRNA for GCAP-II/uroguanylin precursor	0.1147
X63629	H.sapiens mRNA for p cadherin	-
X60489	Human mRNA for elongation factor-1-beta	-
U22055	Human 100 kDa coactivator mRNA	-
M26383	Human monocyte-derived neutrophil-activating protein (MONAP) mRNA	-
L07648	Human MXI1 mRNA	-
R87126	MYOSIN HEAVY CHAIN, NONMUSCLE (Gallus gallus)	-
M91463	Human glucose transporter (GLUT4) gene	-
R44418	EBNA-2 NUCLEAR PROTEIN (Epstein-barr virus)	-
M36634	Human vasoactive intestinal peptide (VIP) mRNA	-
R71875	GLYCOGENIN (Oryctolagus cuniculus)	-
T86473	NUCLEOSIDE DIPHOSPHATE KINASE A (HUMAN)	-
H08393	COLLAGEN ALPHA 2(XI) CHAIN (Homo sapiens)	-
R44301	MINERALOCORTICOID RECEPTOR (Homo sapiens)	-
J05032	Human aspartyl-tRNA synthetase alpha-2 subunit mRNA	-
H77597	H.sapiens mRNA for metallothionein (HUMAN)	-
T48014	HEMOGLOBIN ALPHA CHAIN (HUMAN)	-
U25138	Human MaxiK potassium channel beta subunit mRNA	-
M80815	H.sapiens a-L-fucosidase gene	-
K03460	Human alpha-tubulin isotype H2-alpha gene	-
X68314	H.sapiens mRNA for glutathione peroxidase-GI	-
H20709	MYOSIN LIGHT CHAIN ALKALI, SMOOTH-MUSCLE ISOFORM (HUMAN)	-
H06524	GELSOLIN PRECURSOR, PLASMA (HUMAN)	-
Z49269	H.sapiens gene for chemokine HCC-1	-

Note: - means not being selected in the list

References

[1] Silvio Bicciato, Mario Pandin, Giuseppe Didone, and Carlo Di Bello. Pattern identification and classification in gene expression data using an autoassociative neural network model. *Biotechnology and bioengineering*, 81(5):594–606, 2003.

[2] J. Khan and et al. Classification and diagnostic prediction of cancers using gene expression profiling andartificial neural networks. *Nature Medicine*, 7(6):673–679, June 2001.

[3] M. West and et al. Predicting the clinical status of human breast cancer by using gene expression profiles. *Proc. Natl. Acad. Sci. USA*, 98(20):11462–11467, September 2001.
